# Decoronation: An Alternative Treatment for Replacement Root Resorption

**DOI:** 10.1155/2017/2826948

**Published:** 2017-05-10

**Authors:** Marianne Sala, Asunción Mendoza-Mendoza, Rosa-María Yañez-Vico

**Affiliations:** Department of Stomatology, School of Dentistry, University of Seville, Seville, Spain

## Abstract

**Introduction:**

Ankylosis and disrupted or altered root development are frequent complications associated with intrusive luxation and tooth avulsion lesions. Various forms of treatment have been described according to the severity of the trauma and root development. The literature shows that decoronation is an ideal treatment in cases where replacement resorption occurs.

**Methods:**

Two clinical cases are presented: involving intrusive luxation [15-year-old female patient with an affected maxillary left lateral incisor (2.2)] and a replanted avulsed tooth [8-year-old male patient with avulsion of the right maxillary central incisor (1.1)]; both cases presented advanced root resorption so that decoronation with a prosthetic tooth replacement was decided as the final treatment option.

**Results:**

In the short-term follow-up, patients were asymptomatic and had no functional problems. Radiographs showed that crestal bone height had been preserved.

**Conclusions:**

Preserving the decoronated root in the alveolar process not only helps to maintain bone volume but also enables vertical bone growth and facilitates the future insertion of an implant.

## 1. Introduction

Intrusive luxation is defined as the apical displacement of the tooth into the alveolar bone. It is considered to be one of the most serious types of dental trauma, causing severe affectation to the periodontal ligament, pulp tissue in the root and causing potential alveolar bone loss and growth restriction. In permanent teeth, it is an uncommon injury, representing 0.5–1.9% of all dental injuries [1–5]. There is, however, no consensus about the optimal therapy for minimizing the risk of complications [5–7].

Avulsion, in which the tooth is completely displaced from its socket as a result of trauma, is another type of injury not commonly found in permanent teeth. It represents some 0.5–3% of all dental injuries [8]. Nonetheless, it is one of the most complicated traumatic injuries to treat [9, 10].

In the majority of cases, reimplantation is the treatment of choice, although this cannot always be carried out immediately and is not indicated in certain cases (e.g., in patients with caries and periodontal problems and those who are immunosuppressed) [11, 12]. There is also the danger of replacement root resorption occurring. Management of the dental emergency and the treatment plan are very important for a good prognosis, since the long-term survival rate of reimplanted teeth is low [11, 12].

The objective of reimplanting teeth with immature root development is to facilitate the possible revascularization of the pulp chamber. However, the risk of developing replacement root resorption, or ankylosis, should be weighed against the possibility of revascularization [11, 13].

According to the IADT, signs that suggest a negative prognosis for a replanted avulsed immature permanent tooth with an open apex include [11] symptomatic, excessive, or no mobility (ankylosis) and produce a sharp percussive sound, radiographic evidence of resorption (inflammatory or replacement), and the absence of continued root development.

Until 1984, extraction was the generally accepted option for this kind of complication. Malmgren et al., however, suggested decoronation of the ankylosed tooth as an alternative treatment [12], indicating that, in early mixed dentition, it had to be performed within 2 years of the diagnosis or before the growth spurt, in order to enable the preservation of the surrounding alveolar bone and to prevent infraocclusion and subsequent alterations in smile esthetics [12, 14]. Decoronation can be considered as an ideal treatment in cases where there is replacement resorption. It has been shown that it helps preserve the vestibular-palatal width of the alveolar bone for years, while at the same time allowing for vertical growth, so that it could be used as an alternative treatment in cases with severe root resorption [15, 16]. The aim of the present case report paper is to illustrate the decoronation treatment after two different traumatic injuries, the intrusive luxation and tooth avulsion lesion.

## 2. Clinical Cases

### 2.1. Case  1

**Figure 1:**
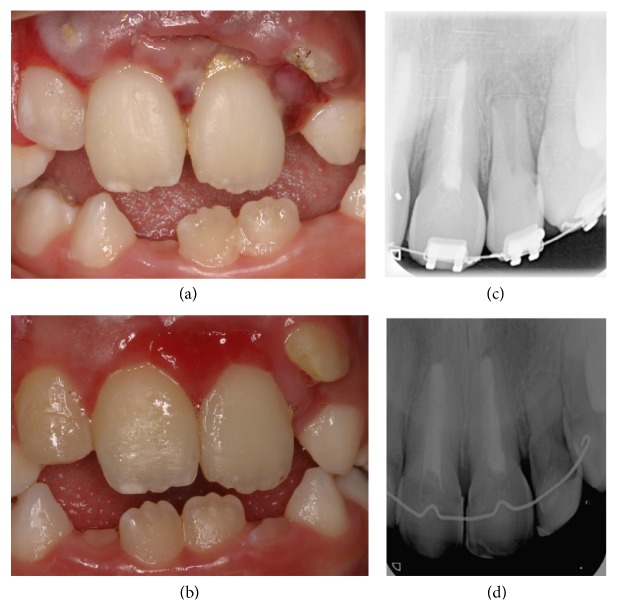
(a) Intraoral photograph of the patient at the clinic following trauma to the 4 incisors, with intrusion of the upper left lateral incisor. (b) Intraoral photograph of the patient following two weeks awaiting spontaneous reeruption of 22, showing insufficient reeruption. (c) Periapical radiographic projection of the orthodontic extrusion carried out after the failure of spontaneous reeruption, with complete extrusion of 22 and root development visibly indicated. (d) Radiographic projection 6 years after the trauma, in which arrested root growth and bone tissue growing inside the root canal can be observed on one of the control periapical radiographs taken after orthodontic treatment.

The first case involved a 15-year-old female patient with replacement root resorption secondary to an intrusion of the maxillary left lateral incisor (2.2) (Figure 1(a)) after a traumatic injury to the 4 upper incisors when she was eight years old, which included soft-tissue laceration. In accordance with the IADT protocol informed consent was obtained and we waited for two weeks, in order to assess whether there had been any tooth movement, indicating the onset of spontaneous reeruption of 2.2 (Figure 1(b)). This did not happen and orthodontic extrusion of the affected tooth was carried out. (Figure 1(c)).

**Figure 2:**
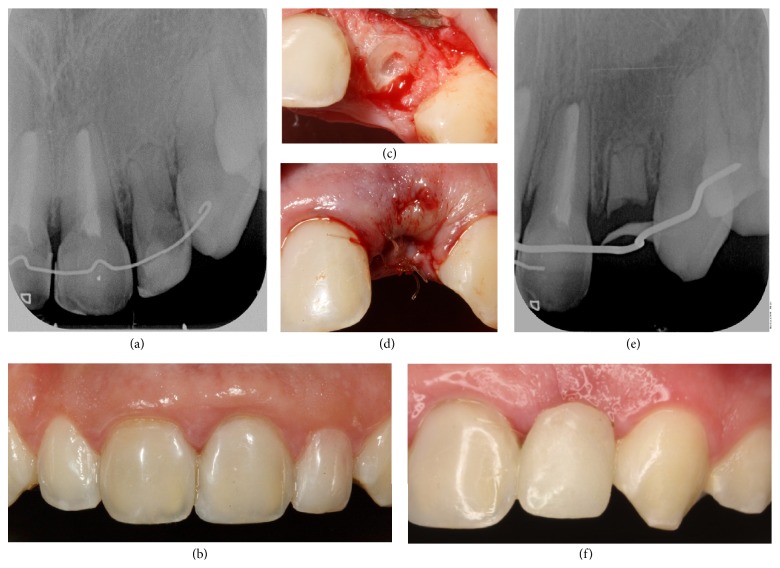
(a) Routine radiographic projection 7 years after the trauma, in which arrested root development can be observed and replacement root resorption in 22 extends from the cervical area down practically the whole of the root. (b) Photograph of the anterior area. Note the clinical aspect of the tooth with suitable aesthetic appearance in harmony with the adjacent teeth. (c) Photograph of 22 during decoronation, after cutting off the crown at the bone margin. (d) Photograph of 22 at the completion of the decoronation procedure, when the full-thickness flap was placed over the alveolus and sutured with interrupted stitches. (e) Radiographic projection of 22 after decoronation, showing the root remaining at the level of the bone crest and the wire for prosthetic rehabilitation. (f) Intraoral photograph of aesthetic rehabilitation achieved via a bridge attached with 0.9 mm wire and a composite pontic, achieving a satisfactory esthetic result.

Clinical and radiographic monitoring was performed after 1 week, 15 days, 1 month, 6 months, and 1 year and then annually up to 5 years. After 6 years, it was observed that the root had stopped growing and that there was bone-like tissue growth in the root canal (Figure 1(d)). After 7 years of clinical and radiological monitoring, it was confirmed that root development had stopped and that resorption had spread from the cervical area to practically the entire root (Figure 2(a)). The clinical appearance of the tooth was completely normal (Figure 2(b)).

When we saw these clinical and radiographic complications, we considered the best treatment option. Since the patient was still growing, it was decided to decoronate the tooth and then fit an adhesive bridge.

After local anesthesia, the decoronation procedure began with the raising of a full-thickness flap to gain direct access to the ankylosed tooth. Then, using a diamond bur, odontosection of the crown was performed above the cementoenamel junction, taking particular care not to leave any enamel residue in the root of the decoronated tooth (Figure 2(c)). After removing the crown, a K-file was used to extract the filling from the root, which was then washed with a saline solution, allowing the canal to fill with blood (if there is no bleeding, it should be stimulated by inserting a K-file through the canal into the apical bone). Finally primary closure was obtained by coronal repositioning of the flap (Figure 2(d)) [12, 17, 18].

We subsequently carried out esthetic rehabilitation using an adhesive bridge with 0.9 mm wire and a composite pontic, taking special care to leave a space of at least 1 mm between the mucous membrane and the pontic to avoid interfering with later alveolar bone remodeling (Figure 2(e)). This gave the patient satisfactory functional and esthetic integrity (Figure 2(f)). Rehabilitation will continue until the patient is fully grown and the necessary conditions for inserting an implant are fulfilled.

### 2.2. Case  2

**Figure 3:**
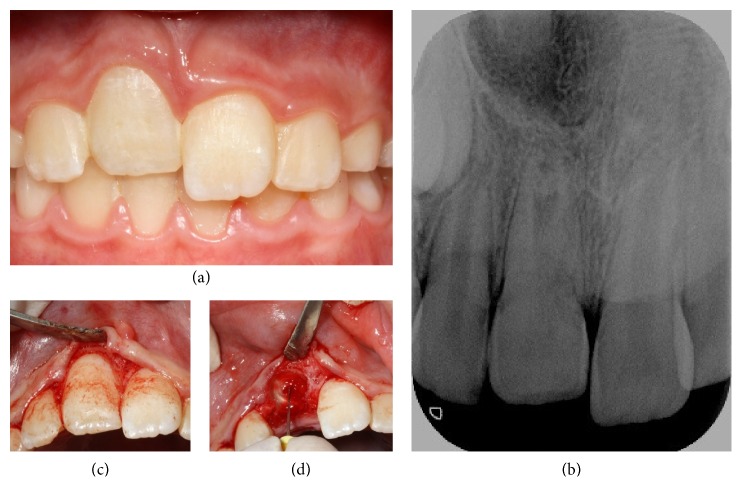
(a) Frontal intraoral photograph of the patient after a trauma to the upper left central incisor [11], which occurred 2 weeks earlier and caused an avulsion that was immediately reimplanted. Observe infraocclusion with respect to 2.1 and 1.2. (b) Periapical control radiograph performed 2 weeks after reinsertion of 11, in which intrusion and ankylosis are apparent. (c) Intraoral photograph during decoronation as the full-thickness flap is raised around the ankylosed tooth. (d) Intraoral photograph during decoronation, as the pulp tissue is removed from the root canal using an endodontic K-file.

Informed consent was obtained from an 8-year-old male patient that came to the clinic after suffering a dental trauma to the right maxillary central incisor (1.1) two weeks earlier that had led to its avulsion, which was reimplanted straightaway after being kept dry for about 40 minutes. Despite poor prognosis reimplantation was conducted in order to preserve vertical and buccolingual bone palatal width of the alveolar crest in a young patient. After the clinical examination, moderate infraocclusion was diagnosed with no discoloration (Figure 3(a)). Radiographs showed arrested root growth and replacement root resorption (Figure 3(b)).

**Figure 4:**
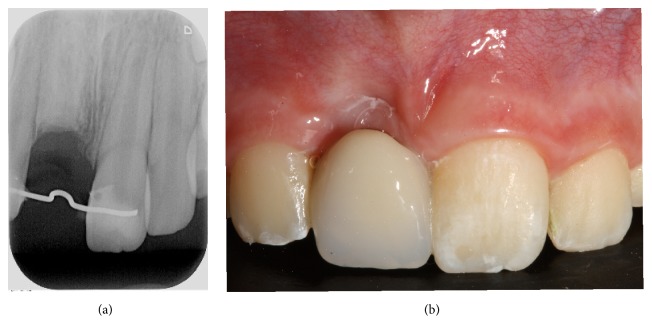
(a) Radiographic projection of 11 showing the root remaining after decoronation and the wire for prosthetic rehabilitation. Note the absence of the periodontal ligament space. (b) Intraoral photograph showing aesthetic rehabilitation using a bridge attached with a 0.9 mm wire and a composite pontic, giving an acceptable aesthetic appearance and leaving a 2 mm space between the mucous membrane and the pontic so as not to interfere with bone remodeling.

After several clinical and radiological reviews, we considered the best time for carrying out decoronation, which was eventually performed 2 years after diagnosis, when the patient was 10 years old. The decoronation procedure followed the methodology described for case 1 (Figures 3(c) and 3(d)). Afterwards, provisional aesthetic rehabilitation was carried out, using an adhesive bridge, which will remain until an implant can be inserted (Figures 4(a) and 4(b)).

## 3. Discussion

According to Andreasen and Pedersen [19], any damage to the affected tooth or factors associated with the injury can influence the prognosis for healing. These factors include age of the patient, stage of root formation, tooth type, potential crown fractures, extent of displacement, presence or absence of gingival laceration, and the number of intruded teeth. The stage of root development is considered to be one of the most important factors [19, 20].

Andreasen et al. [21] came to the conclusion that the possibility of spontaneous reeruption had to be allowed for in intruded teeth with immature root development. In teeth with mature root development (12–17 years old), however, spontaneous reeruption might be allowed for, while in patients with mature roots (>17 years), orthodontic or surgical repositioning should be performed. Other authors, such as Wigen et al. [22], recommend a period of observation for patients between 6 and 12 years old, irrespective of root development, while waiting for spontaneous reeruption. What is clear is that treatment should be directed towards eliminating or reducing secondary complications of the injury. The literature describes that, in such cases, the new eruption process may last from 1 to 8 months [23, 24]. In case 1, we followed the IADT protocol and waited two weeks for spontaneous reeruption, since the tooth root was immature and ought to have retained its potential for eruption. When we carried out clinical and radiological controls after 2 weeks, no tooth movement was observed in the alveolar process and so we opted for orthodontic traction.

Orthodontic extrusion is one of the alternative therapies of choice for intruded permanent teeth, because it allows for the remodeling of the bone and the periodontal apparatus [25, 26]. The intruded tooth can be orthodontically repositioned for endodontic treatment later, if necessary, within a time scale that may vary between 2 and 3 weeks following activation [27].

Although both spontaneous reeruption and orthodontic repositioning cause less damage to the surrounding tissues, there is no general agreement about the choice of one or other treatment [21]. Some authors [28] have recommended that only in cases where there is very slight intrusion should we wait and see if there is spontaneous reeruption. Others have suggested that monitoring should continue until the patient is 17 years old, independently of the intrusion [21]. If reeruption does not occur, forced orthodontic eruption may be applied, which is why, in case 1, after waiting for two weeks and failing to observe any reeruptive movement, we performed orthodontic traction as the second treatment option [27]. However, neither of these options prevented the interrupted root development and subsequent ankylosis (Figure 1(c)).

When the periodontal ligament (PDL) is damaged, there are 4 possible sequelae for healing or nonhealing. One of these is replacement root resorption, or ankylosis, or osseous replacement resorption. Histologically speaking, these are areas where the dentin can be observed as fused with the surrounding bone, with resorption pits where the multinucleated osteoclasts are actively resorbing the dentin, and the PDL and cement have been lost. Radiographically, the root appears to merge with the surrounding bone, with the complete absence of the PDL [29–32]. Both our clinical cases developed this complication. In case 1, six years after the initial trauma, root resorption with bone-like tissue growth in the root canal was observed on a radiograph at one of the patient's annual check-ups for orthodontic treatment. In case 2, ankylosis was observed on the control radiograph (Figure 3(a)) a few months after the trauma and as a direct result of it. The main challenge presented by this kind of resorption is that there is no way of stopping it once it has started. Endodontic treatment is not recommended for ankylosis unless there are signs and symptoms consistent with pulp necrosis [29–32].

Teeth that are reimplanted in children who develop ankylosis after a trauma can be used to preserve the height/width of the alveolar bone [12]. The mechanism of action of this beneficial effect could be associated with an active periosteum forming over the resorbed root, which allows the root to serve as a scaffold for alveolar bone neogenesis similar to that of the adjacent erupting tooth [17].

The success of this technique has been clinically described in 77 patients with tooth ankylosis after avulsion [33]. In almost half of the patients, alveolar bone was detected 1.5 years after decoronation; in 61%, remnants of the root were still present. In those patients who had undergone this procedure during the pubertal growth spurt, no infraposition of the bone segment was seen during monitorization following the procedure. However, vertical bone level increased and the patients maintained the alveolar bone in the labiopalatal dimension in the long-term follow-up, as learned from excellent reports [23, 34, 35].

In the clinical cases presented, we show two profiles of different ages in which we opted for decoronation as a treatment for ankylosis. In this regard, two successive studies presented by Malmgren et al. [12, 17] have suggested that, in early mixed dentition (7–10 years), decoronation should be performed within 2 years of the diagnosis or before the growth spurt. In case 2, two and a half years after the initial trauma and 2 years after the first sign of ankylosis, we decided to perform decoronation before the following growth spurt because of the moderate infraposition of 1.1 and also to prevent the bone defect from becoming more apparent. In late mixed dentition (10–12 years), discretion should be used, depending on each case, and intervening sooner rather than later. If ankylosis occurs during the growth spurt, which implies infraocclusion, decoronation should be carried out as soon as the problem is diagnosed. This highlights the critical importance of clinical and radiographic monitorization in such type of traumatic cases. In case 1, decoronation was performed when the patient was 15 years old (during pubertal growth), to take full advantage of the several millimeters increase in the vertical bone level and the preservation of the buccopalatal width of the alveolar crest. Surgical intervention for decoronation demands excellent management of children behavior especially in young children, that is, case 2 (8 years old); nevertheless this type of procedure does not differ excessively from other therapeutic options in terms of patients' perceived anxiety or pain [36, 37].

This procedure enables us to avoid a later costly invasive surgical procedure to augment the alveolar ridge. However it is still a surgical intervention, which can pose a challenge for young children. A “temporary” replacement for the missing teeth will also be required for a long period [29]. In spite of these disadvantages, there are good indications that the bone that forms in the replacement root resorption area is of good quality. Placing implants in sockets when the individual is fully developed is a relatively easy and uncomplicated matter [12]. The main limitations of the present report reside in its methodological nature that is just a case report study so no direct evidence based conclusions might be derived from this illustrative report.

## 4. Summary

Of the possible options, decoronation is an alternative treatment that offers a good clinical outcome. It has been shown that if this procedure is carried out at the right time, the buccopalatal alveolar dimensions can be preserved for years, while at the same time allowing for extra vertical growth, which makes restoration possible later with an implant.

This is an easy safe procedure that represents a conservative approach to extracting ankylosed teeth when compared to a major surgical intervention. Solid studies are required in the future to enable us to determine how predictable and successful decoronation is in the long-term as a treatment after ankylosis in the anterior teeth.
